# Phosphorus availability and leaching losses in annual and perennial cropping systems in an upper US Midwest landscape

**DOI:** 10.1038/s41598-021-99877-7

**Published:** 2021-10-13

**Authors:** Mir Zaman Hussain, Stephen K. Hamilton, G. Philip Robertson, Bruno Basso

**Affiliations:** 1 W.K. Kellogg Biological Station, Michigan State University, Hickory Corners, MI 49060 USA; 2 Great Lakes Bioenergy Research Center, Michigan State University, East Lansing, MI 48824 USA; 3 Department of Integrative Biology, Michigan State University, East Lansing, MI 48824 USA; 4 Cary Institute of Ecosystem Studies, Millbrook, NY 12545 USA; 5 Department of Plant, Soil, and Microbial Sciences, Michigan State University, East Lansing, MI 48824 USA; 6 Department of Earth and Environmental Sciences, Michigan State University, East Lansing, MI 48824 USA

**Keywords:** Biogeochemistry, Environmental sciences, Hydrology

## Abstract

Excessive phosphorus (P) applications to croplands can contribute to eutrophication of surface waters through surface runoff and subsurface (leaching) losses. We analyzed leaching losses of total dissolved P (TDP) from no-till corn, hybrid poplar (*Populus nigra* X *P. maximowiczii*), switchgrass (*Panicum virgatum*), miscanthus (*Miscanthus giganteus*), native grasses, and restored prairie, all planted in 2008 on former cropland in Michigan, USA. All crops except corn (13 kg P ha^−1^ year^−1^) were grown without P fertilization. Biomass was harvested at the end of each growing season except for poplar. Soil water at 1.2 m depth was sampled weekly to biweekly for TDP determination during March–November 2009–2016 using tension lysimeters. Soil test P (0–25 cm depth) was measured every autumn. Soil water TDP concentrations were usually below levels where eutrophication of surface waters is frequently observed (> 0.02 mg L^−1^) but often higher than in deep groundwater or nearby streams and lakes. Rates of P leaching, estimated from measured concentrations and modeled drainage, did not differ statistically among cropping systems across years; 7-year cropping system means ranged from 0.035 to 0.072 kg P ha^−1^ year^−1^ with large interannual variation. Leached P was positively related to STP, which decreased over the 7 years in all systems. These results indicate that both P-fertilized and unfertilized cropping systems may leach legacy P from past cropland management.

## Introduction

Phosphorus (P) is a key limiting nutrient for primary producers both on land and in water. In unfertilized soils, concentrations of soluble P tend to be low due to the immobilization of P in organic and inorganic forms that are less available to plants. Therefore, P fertilization is commonly needed to meet crop demand, often at high rates to build up “insurance P” in the soil, and over the long term this can lead to saturation of soil P sorption capacity and subsequent higher P concentrations in soil solution^1,2^. Excess P can be leached with percolating water to groundwater, and particularly in sandy soils or where there is tile drainage, some can reach surface waters, thereby posing a significant risk for eutrophication^3^. As a result of P applications in excess of harvest removal over long periods, many agricultural soils have accumulated “legacy P,” some of which can be subject to hydrologic export for years to decades after the excess inputs cease^4–6^.

Nutrient export from croplands in the US Midwest is a particular problem with annual crops and especially corn (*Zea mays* L.) because of its high nutrient demands and the considerable landscape water movement that occurs outside of the growing season in temperate climates. The United States contributes 36% of global corn grain production, with much of this production in the US Midwest^7^. Increasing demand for grain ethanol production has led to an increase in corn acreage by roughly 10 percent (7.2 million acres) between 2000 and 2009^8^. The increasing cultivation of corn has been linked to greater risk of P export to rivers and coastal marine waters^6,9,10^.

Dissolved forms of P and particularly orthophosphate (H_2_PO_4_^−^) cause the eutrophication of water bodies due to their availability for biological uptake^11^. Dissolved P concentrations exceeding ~ 0.02 mg L^−1^ are considered sufficient for eutrophication in surface waters in agricultural regions of the US Midwest^12,13^ and eastern Canada^14,15^ as well as in many other regions of the world^3,16^.

Mitigation of P leaching from agricultural soils is desirable, and one approach is growing and harvesting plants that accumulate P. Several perennial grasses^17,18^ as well as annual crops like sorghum^19^ have been suggested. Due to their low fertilization requirements, perenniality, and high biomass production, grasses like miscanthus and switchgrass^20–22^ as well as short-rotation poplar^23–25^ might also reduce nutrient leaching while offering other environmental benefits^26,27^. Most studies of nutrient leaching from these kinds of cropping systems have examined nitrogen, however; there are relatively few studies of P leaching from perennial crops^24,28,29^.

Here we present 7 years of measurements of dissolved phosphorus concentrations in soil water beneath the root zones of conventional P-fertilized corn (Table 1); perennial grasses including miscanthus (*Miscanthus giganteus* J.M. Greef & Deuter ex Hodkinson & Renvoiz), switchgrass (*Panicum virgatum* L.), native grasses, and restored prairie; and poplar trees (*Populus nigra* L. X *P. maximowiczii* A. Henry ‘NM6’). All crops but corn were grown without P fertilization in close proximity on former cropland in Michigan, USA. Leaching rates were calculated by multiplying soil water P concentrations by modeled rates of water drainage. Bray-1 soil test P (STP) was measured each autumn. We compare P leaching losses and soil test P concentrations among the perennial and annual cropping systems across 7 years spanning establishment and stabilization of the perennial cropping systems and encompassing a wide range of climate variability. We hypothesized that P leaching rates would be much lower in the unfertilized perennial crops than in corn, and that if legacy P is important, P leaching rates and soil test P concentrations should decrease significantly over time.

**Table 1:** Nitrogen (N) and phosphorus (P) fertilizer applications (kg ha^−1^) to the cropping systems.

	Corn		Switchgrass		Miscanthus		Native grasses		Poplar		Restored prairie	
	N	P	N	P	N	P	N	P	N	P	N	P
Crop-year	**kg ha**^**−1**^											
2009	158	13	0	0	77	0	0	0	0	0	0	0
2010	176	13	56	0	56	0	56	0	0	0	0	0
2011	172	13	56	0	56	0	56	0	157	0	0	0
2012	174	13	56	0	20	0	20	0	0	0	0	0
2013	173	13	57	0	57	0	57	0	0	0	0	0
2014	169	13	57	0	57	0	57	0	0	0	0	0
2015	172	33	57	0	57	0	57	0	0	0	0	0
Total	1194	111	339	0	30	0	303	0	157	0	0	0

## Results

### Climate, hydrology, growing season length, and crop productivity

Air temperature recorded from a nearby weather station averaged 9.3 °C (mean for 2009–2015), which was somewhat warmer than the long-term average (1988–2015: 9.1 °C). Annual precipitation based on crop-years (defined from the date of planting or leaf emergence in a given year to the day prior to planting or emergence in the following year) was lowest in crop-year 2009 (725 mm), well below the long-term average (1988–2015: 915 mm), but in the other crop-years it was close to or above the long-term average (mean for 2010–2015: 1000 mm) (Table 2). However, May–Sep growing-season precipitation was considerably lower in 2012 (227 mm) than in 2009 (358 mm) or in the other years of the study, which in combination with high temperatures in the spring and summer of 2012 resulted in a severe agricultural drought that reduced crop yields in the region.

**Table 2:** Precipitation (precip, mm) and mean modeled drainage (mm) by growing season (May–Sep) and non-growing season (Oct–Apr). Annual precipitation is given for the approximate crop-year periods (May–April). Drainage data are the mean ± s.e. of across all six cropping system systems, which were not statistically distinct.

Year	Growing season		Non-growing season		Annual	
	Precip	Drainage	Precip	Drainage	Precip	Drainage
**mm**						
2009	358	79 ± 6.6	368	95 ± 5.1	726	174
2010	568	149 ± 11.3	465	149 ± 18.6	1033	298
2011	556	175 ± 10.6	569	216 ± 15.3	1125	391
2012	227	66 ± 12.1	694	309 ± 21.8	921	375
2013	446	96 ± 22.6	499	239 ± 13.0	945	335
2014	473	94 ± 7.2	447	175 ± 5.5	920	269
2015	726	307 ± 31.8	448	166 ± 10.5	1174	473
Mean	479	138	499	193	978	331

Drainage rates simulated by SALUS over the 7 years did not differ significantly among cropping systems (Table 3). The mean annual drainage was 331 mm, which was 34% of mean annual precipitation, and on average 42% of the annual drainage occurred in the Oct–Apr non-growing season (Table 2). Interannual variation in drainage rates mirrored that of precipitation. The modeled drainage rates in this study are consistent with rates for this site reported in a 6-year study using large-diameter monolith lysimeters^30^.

**Table 3:** Maximum aboveground biomass, growing season length, annual drainage, Bray-1 soil test P, total dissolved P (TDP) leaching rates, and volume-weighted mean P concentrations (means ± s.e.) across the seven crop-years (2009–2015) in each cropping system. The p values indicate which variables show significant differences among the systems.

Cropping system	Max. biomass (Mg ha^−1^)	Growing season length (days)	Drainage (mm)	Bray-1 soil P (mg P kg^−1^)	Bray-1 soil P (kg P ha^−1^)	TDP leached (kg P ha^−1^ year^−1^)	Volume-wtd mean concentration (mg P L^−1^)
Corn	20.4 ± 0.9	180	360 ± 37.2	34.7 ± 9.9	156 ± 44	0.035 ± 0.01	0.009 ± 0.003
Switchgrass	7.7 ± 0.9	185	310 ± 33.0	21.3 ± 3.2	95 ± 14	0.048 ± 0.03	0.014 ± 0.01
Miscanthus	23.9 ± 2.4	190	342 ± 49.2	32.6 ± 3.5	147 ± 16	0.040 ± 0.02	0.013 ± 0.01
Native grasses	8.1 ± 0.9	187	303 ± 32.7	21.1 ± 1.2	95 ± 5.5	0.060 ± 0.03	0.020 ± 0.01
Poplar	30.8 ± 1.9	207	315 ± 28.0	45.6 ± 6.6	121 ± 15.5	0.047 ± 0.02	0.016 ± 0.01
Restored prairie	6.8 ± 0.8	179	358 ± 26.8	27.0 ± 3.5	205 ± 30	0.072 ± 0.04	0.020 ± 0.01
p value	< 0.05		> 0.05	< 0.05	< 0.05	> 0.05	> 0.05

The length of the growing-season averaged 180 days for corn, 179–190 days for the grasslands, and 207 days for poplar (Table 3), and lasted approximately from May–Sep. Maximum aboveground biomass (Mg ha^−1^) averaged across the 7 years (including the four replicate plots) differed significantly (p < 0.05) among cropping systems (Table 3). The maximum aboveground biomass was highest in poplar, miscanthus, and corn, while it was lowest in switchgrass, native grasses, and prairie. The mean maximum aboveground biomass of corn was lower than poplar and miscanthus, but higher than the other perennial systems. Poplar, which was harvested during the 2013–14 winter, had an annual cumulative increment up until that harvest, after which a diversity of weeds as well as coppicing poplar stems occupied the plots.

### Phosphorus leaching losses

**Figure 1:**
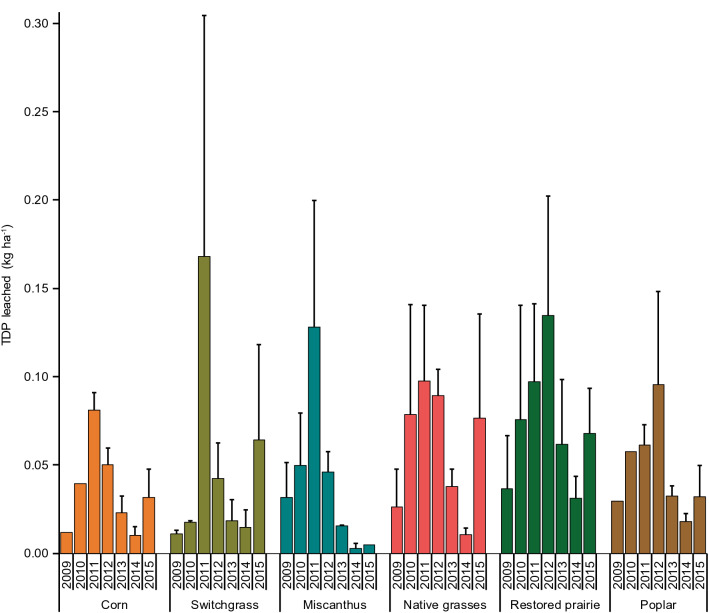
Annual leaching losses of total dissolved P (kg ha^−1^) from the different cropping systems. Each bar shows the standard error of the mean of four replicate plots. In some crops during the 2009 and 2010 crop-years, data were available for only one or two plots. The cropping-system means grouped across years are not significantly different (p > 0.05) in pairwise comparisons as determined by the Tukey honest significant difference test. Years refer to crop-years that extend from planting or emergence of the crop in the year indicated through the ensuing year until the next year’s crop planting or emergence.

Across all seven crop-years, P leaching rates were statistically indistinguishable (p > 0.05) among the cropping systems, averaging between 0.035 and 0.072 kg P ha^−1^ year^−1^ (Table 3), and the rates showed substantial interannual variability in every system (Fig. 1). Four of the six cropping systems had the highest leaching rates in 2011, whereas the restored prairie and poplar systems leached more P in 2012; those 2 years differed markedly in precipitation, with normal precipitation in the May–Sep growing season of 2011 and a severe growing-season drought in 2012 (Table 2). Leaching occurred mostly outside the growing season (Oct–Apr), and rates were significantly correlated with total non-growing-season precipitation and drainage (Pearson’s R = 0.55 and 0.32, respectively). Using corn and switchgrass as examples of an annual fertilized and perennial unfertilized crop, respectively, about 65% of the annual P leaching occurred in periods that could not be sampled (Dec–Mar).

**Figure 2:**
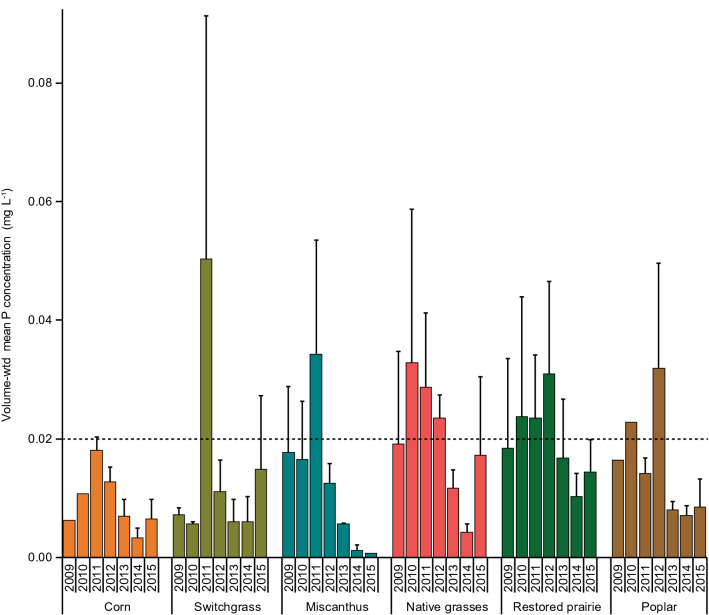
Volume-weighted mean concentrations (mg L^−1^) of total dissolved phosphorus in leachate from the different cropping systems collected with soil water samplers at ~ 1.2 m depth. Each bar shows the standard error of the mean of four replicate plots. In some crops during the 2009 and 2010 crop-years, data were available for only one or two plots. The cropping-system means grouped across years are not significantly different (p > 0.05) in pairwise comparisons as determined by the Tukey honest significant difference test. Dashed line indicates the critical P concentration above which surface waters are prone to eutrophication^3,16^. Years refer to crop-years that extend from planting or emergence of the crop in the year indicated through the ensuing year until the next year’s crop planting or emergence.

Similarly, volume-weighted mean TDP concentrations over the seven crop-years were statistically indistinguishable (p > 0.05) among all of the cropping systems, averaging between 0.009 and 0.020 mg L^−1^, but the concentrations showed substantial interannual variability in every system (Table 3, Fig. 2). In at least 1 year in every cropping system except corn, the volume-weighted TDP concentration exceeded the levels where eutrophication of surface waters is frequently observed (> 0.02 mg L^−1^)^3,16^. Corn was the only system that was annually fertilized with P (Table 1), but it tended to have the lowest overall rates of P leaching, albeit not significantly different overall from the other systems.

### Soil test phosphorus

**Figure 3:**
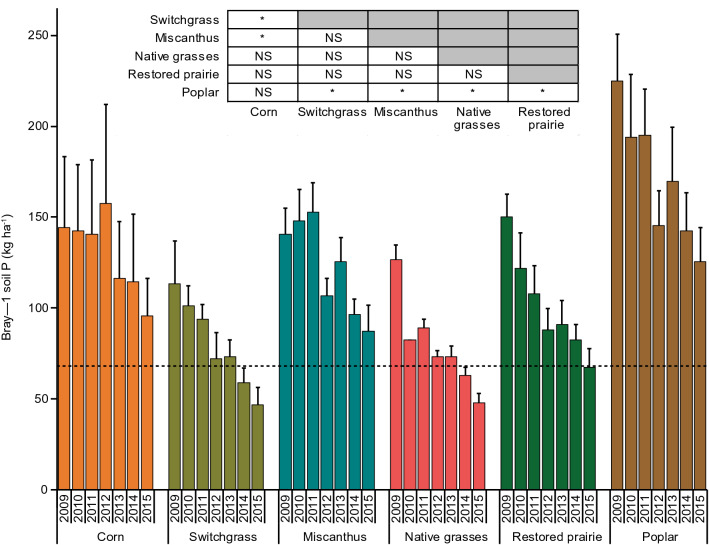
Bray-1 soil test phosphorus in the upper 25 cm of soil in the different cropping systems. Each bar shows the standard error of the mean of four replicate plots. The inset shows which system means grouped across years are significantly different (*) in pairwise comparisons as determined by the Tukey honest significant difference test (α = 0.05; NS = not significantly different). Bold dashed line indicates critical lower threshold of soil test phosphorus recommended for corn production (68 kg P ha^−1^, equivalent to 15.0 mg kg^−1^).

**Figure 4:**
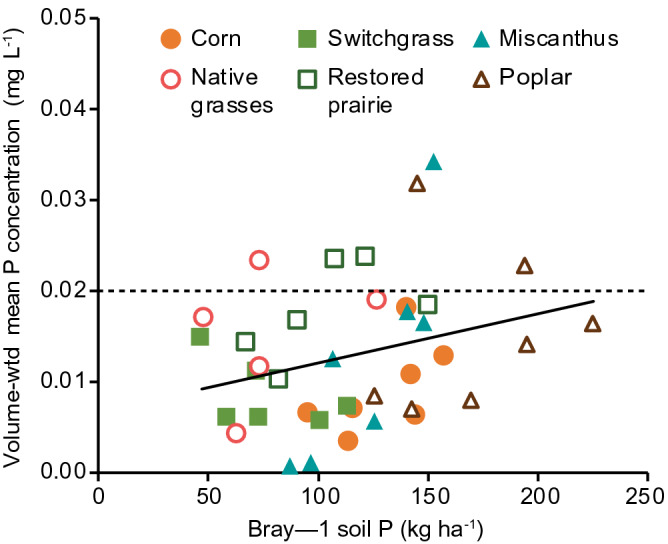
Relationship between Bray-1 soil test phosphorus and annual volume-weighted mean total dissolved phosphorus concentrations in leachate. The correlation is significant for all data combined (p < 0.05; r = 0.34). Dashed line indicates the critical total phosphorus concentration above which surface waters are prone to eutrophication^3,16^.

Initial Bray-1 STP (0–25 cm depth) concentrations (kg P ha^−1^ soil) during the 2009 crop-year averaged 173 in corn, 136 in switchgrass, 168 in miscanthus, 151 in native grasses, 180 in prairie, and 270 in poplar, respectively (Fig. 3). Mean STP concentrations over the 7-year period varied significantly (p < 0.05) among cropping systems, being highest in poplar (205 kg P ha^−1^) and lowest in switchgrass and native grasses (95 kg P ha^−1^) (Fig. 3, Table 3). Although declining over time (see below), the mean STP concentration across the 7 years (137 kg P ha^−1^) was well above the minimum agronomic P (68 kg P ha^−1^) recommended for corn production in the US Midwest^31^. The annual volume-weighted TDP concentrations in soil solutions were positively related to STP concentrations when data from all cropping systems and years were combined (r = 0.34, p < 0.05, Fig. 4).

**Figure 5:**
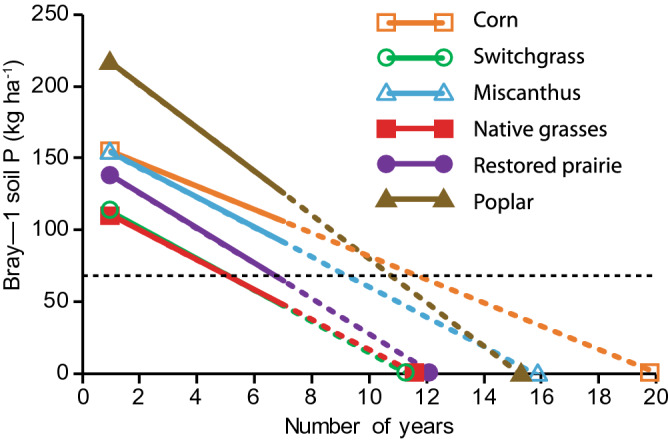
Changes in Bray-1 soil test phosphorus concentration over the years under continuous corn, switchgrass, miscanthus, native grasses, prairie, and poplar. Linear relationships are shown by colored bold regression lines (y = − 2.1607x + 43.357, r^2^ = 0.65 for corn; y = − 2.9018x + 32.92, r^2^ = 0. for switchgrass, y = − 2.7679x + 43.75, r^2^ = 0.74 for miscanthus; y = − 2.7768x + 32.25, r^2^ = 0.83 for native grasses; y = − 3.267x + 40.071, r^2^ = 0.91 for prairie; y = − 4.0625x + 61.857, r^2^ = 0.86 for poplar); while colored dashed regression lines represent predicted change in the STP concentrations when trends beyond 7 years are extrapolated. Black dashed line indicates the critical lower threshold of soil test phosphorus to satisfy crop requirements (68 kg ha^−1^, equivalent to 15.0 mg kg^−1^).

STP concentrations decreased over the 7 years in all cropping systems, including in corn where P fertilizer was annually applied. By the 7th year, STP concentrations in the switchgrass and native grass systems fell below the minimum recommended agronomic P of 68 kg P ha^−1^ for corn production (Fig. 3). Compared with initial STP concentrations, concentrations after 7 years had decreased to 114 kg P ha^−1^ in corn (34% reduction), to 104 kg P ha^−1^ in miscanthus (38% reduction), to 81 kg P ha^−1^ in prairie (55% reduction), and to 150 kg P ha^−1^ in poplar (44% reduction), in all four cases remaining above the minimum recommended agronomic P level for corn. In contrast, STP concentrations in the switchgrass and native grasses systems fell below the threshold P level by year 7. Extrapolation of these decreases suggests that all cropping systems would fall below the P optimum within 6–13 years of establishment (Fig. 5).

### Phosphorus in lakes, streams, and groundwater

**Figure 6:**
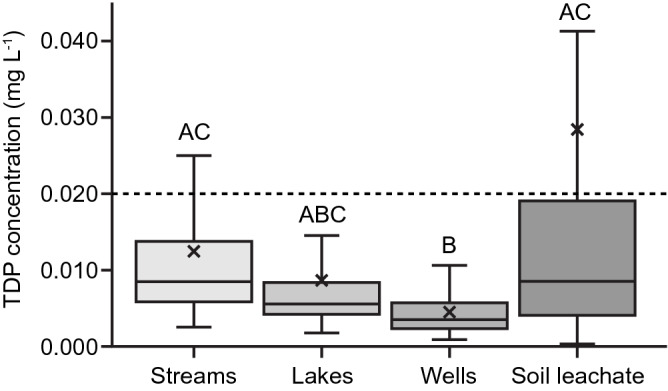
Total dissolved phosphorus concentrations (mg L^−1^) in water samples collected from lakes, streams, and groundwater wells compared to total dissolved phosphorus (TDP) concentrations in soil leachate sampled from different cropping systems. TDP was measured from 2009–2016 in lakes (n = 597), streams (n = 283), wells (n = 33), and soil water leachates (this study; n = 899). Horizontal lines within each box indicate the median while the asterisk show the mean. The upper and lower ends of the boxes denote the 25th and 75th percentiles, while the whiskers indicate 10th and 90th percentiles. Dashed line indicates the total P concentration above which surface waters are prone to eutrophication^3,16^ When combination of water bodies shares a similar letter, the means grouped across years are not significantly different as determined by the Tukey honest significant differences post-hoc test (α = 0.05). The Tukey–Kramer method was used to make pairwise comparisons among groups because of the unequal sample sizes.

The volume-weighted mean TDP concentrations in soil leachates were higher than TDP concentrations in most local streams, lakes, and groundwater wells (Fig. 6). Streams in the area carry higher TDP concentrations than lakes, and both tend to be higher in TDP than deeper groundwater from wells, but in all cases most of the concentrations are well below the levels where eutrophication of surface waters is frequently observed (> 0.02 mg L^−1^).

## Discussion

Results do not support our hypothesis that P leaching rates would be lower in the unfertilized perennial crops than in corn-in fact, there were no overall differences in P leaching among cropping systems when considering the entire 7-year period, and there was a tendency for corn to show lower leaching than the perennial crops despite corn’s annual P fertilization, perhaps because corn was able to more efficiently utilize the soil P (Fig. 1). Interannual variation in P leaching rates was large, yet in contrast to STP there was not a consistent decreasing trend over time, suggesting no direct relation with STP over the range of STP concentrations measured in this study.

The observed annual rates of P leaching (< 0.2 kg P ha^−1^ year^−1^) reported here for our well-drained, sandy loam soils are consistent with other studies of cropping systems on mineral soils, which have generally found rates of < 1 kg P ha^−1^ year^−1^^32^, although some of the rates in our study (corn: 0.04 kg P ha^−1^; perennial grasses and poplar: ~ 0.05 kg P ha^−1^, Table 3, Fig. 1) are on the low end of the range. For example, other studies of P leaching conducted in moderately to poorly drained soils have reported rates of 0.03–1.09^33^, 0.10^34^ and 0.05–1.0 kg P ha^−1^^13^ in annual crops and 0.25–4.39 kg P ha^−1^ in perennial crops^35^. Considering that our soils are coarser in texture with higher sand content than many of the other locations where P leaching has been studied, leaching rates on the higher end of the range may have been expected, but other factors such as fertilization rates and management history may also explain the variation in rates among different settings. Also, research on P leaching might tend to be conducted in locations where it is known or perceived to be a problem, resulting in a bias toward systems with higher P losses.

As noted earlier, some other studies have found similar rates of P leaching between corn and perennial cropping systems. Brye et al.^28^ reported no difference in rates of P leaching under corn and prairie systems grown on silt loam soil in south central Wisconsin (USA) over a 5-year period. Likewise, Daigh et al.^29^ observed no significant differences in P leaching between corn and prairie systems grown on fine-loamy soil in Iowa (USA) over a 4-year period. Comparing different perennial crops in Florida (USA), Silveira et al.^18^ found no difference in leachate P concentrations among sugarcane, stargrass, and switchgrass cropping systems grown on manure-enriched Spodosols. These observations suggest a high degree of buffering of P leaching by soil P stocks influenced by long-term land use, as noted by other studies^36–40^.

Any infiltration that occurs in mid-winter was not sampled by our study, like most other studies conducted in climates where soils tend to freeze over winter, precluding sample collection. In our study, we used the mean TDP concentrations measured before and after the mid-winter period in combination with infiltration estimated by the SALUS model over that period to estimate P leaching. Considering that ~ 65% of the estimated annual P leaching occurred during the mid-winter period, this represents a major source of uncertainty in our estimates. However, we have no reason to expect that P leaching would be particularly higher or lower in mid-winter compared to the closest late autumn or early spring sampling dates, nor to expect crop-specific differences in P leaching during mid-winter when all crops are dormant.

The quantity of annually leached P observed in our study was an insignificant fraction (< 1%) of total soil phosphorus (measured as STP in the surface soil) (Fig. 3), and also an insignificant fraction of the P added as fertilizer to the corn system (Table 1). Although such losses may therefore seem agronomically inconsequential, they may nonetheless contribute to the eutrophication of sensitive surface waters, as discussed later.

That the unfertilized perennial cropping systems leached as much or more P than the fertilized corn system suggests P loss from a large soil stock of legacy P in these systems. High initial STP concentrations (> 130 kg P ha^−1^ or equivalent to > 30 mg P kg^−1^) (Fig. 3) may reflect decades of cropping and manuring^28,36,37^ even though loam soils at our site (up to 76% sand) typically have a low P sorption capacity compared to finer-textured soils^41–43^. As noted in the “Methods” section, available records of P fertilization as manure and inorganic fertilizer date back to 2003 and indicate fairly high P application rates over the 5 years preceding the start of the experiment, although they do not explain the spatial variability we observed for STP because the field was mostly fertilized uniformly. Despite high initial STP, the observed decreases in STP over time suggest that 7 years of perennial crop production and harvest, particularly in the switchgrass and native grass systems, have drawn down much of the legacy P. Preliminary P budgets of these systems including poplar suggest that the removal of P by harvest roughly corresponded with decreasing STP concentrations in the perennial cropping systems, although P fertilization approximately counterbalanced the harvest P removal in corn (data not shown). Our measured STP concentrations reflect only the upper 25 cm of soil and are thus an underestimate for STP in the entire soil profile above the depth of soil water collection, although other studies in cropping systems have found the highest STP concentrations towards the soil surface^18,44^, and the lower part of the profile at our site is more dominated by sand that likely has a low P-binding capacity.

In addition to harvest, the decrease in STP in the surface soil of the perennial cropping systems may be attributable to incremental increases in P stored in roots of the perennial crops and, additionally in the case of poplar that was not harvested, in aboveground biomass^25,45–47^. Despite the considerable decrease in STP in the perennial cropping systems, three of those five systems remained above the minimum recommended agronomic P level for corn by 7 years after planting, although the time for depletion of legacy P depends on the starting STP concentrations, which were variable, as well as the rate of decline (Fig. 5).

On average, the volume-weighted mean concentrations of TDP in soil leachates were higher than in local groundwater wells, and tended to be higher than in local lakes and streams (Fig. 6). The difference between P concentrations in leachates and groundwater drawn from this glacial aquifer could reflect a combination of (1) P retention in the glacial deposits below the root zone by sorption to minerals^1^, (2) that only about a third of the local landscape is in cropland now^48^, and (3) time lags for contamination of the aquifer due to its long water turnover time^49^.

Many cases of P eutrophication of surface waters have been documented in heavily farmed watersheds in the broader region^13,15,34^ and elsewhere^2,6,50^ Primary production in lakes and streams in this region tends to be strongly P-limited, and thus P concentrations in the water column are usually lower than in source waters^51^. The growth of algae and vascular plants in these ecosystems is naturally sensitive to increased watershed inputs of P. Relative to the lower P concentrations in lakes and streams in the region, the volume-weighted mean concentrations of P leached from cropping systems suggest that leachate could carry significant amounts of P to surface waters. Transport of P from croplands to surface waters is most likely where there is overland flow, especially when it transports particulate matter eroded from soils, or where subsurface flow through preferential flow pathways or artificial drainage systems bypasses the potential for P retention in underlying soils^1,49^. In many areas of the Midwest US as well as other regions, agricultural fields have artificial drainage systems (e.g., tile drains) that reroute leached P from the rooting zone to surface waters. The conclusions reached in our study may apply as well to tile-drained fields because the drain tiles are typically at a similar depth as our soil water samplers.

We found drainage water P concentrations in some years (Table 3, Fig. 2) that approached or exceeded the total P concentration above which eutrophication of surface waters is frequently observed (> 0.02 mg L^−1^^3,16^. Other studies have reported a similar range of P concentrations in leachate from corn and perennial systems in the US Midwest^28,29^, whereas much higher leachate P concentrations have been found for some cropping systems elsewhere in the US (e.g., 0.05–0.6 mg L^−1^^52^; 0.1–0.7 mg L^−1^^53^; 0.6–1.3 mg L^−1^^17^). Together with these studies, our data suggest that leaching of P from croplands, though low relative to soil P stores, can pose a potential concern for surface water quality.

## Conclusions

Results from this 7-year study show that soils can continue to leach P even under non-fertilized crops that are significantly drawing down soil P stocks. Modest P fertilization of corn did not result in a detectable increase in P leaching compared to the unfertilized perennial crops. The lack of differences due to cropping system type (annual vs. perennial) or fertilization practices suggests a high degree of buffering by soil P stocks accumulated during past agricultural activities (i.e., legacy P). Leaching of P over the 7 years of this study represented only a small fraction of soil P stocks and can readily be compensated by fertilization. Nonetheless, the export of leached P can potentially cause eutrophication of P-limited surface waters typical of this region and thus P leaching is a concern for water quality protection.

## Materials and methods

### Experimental details

The Biofuel Cropping System Experiment (BCSE) is located at the W.K. Kellogg Biological Station (KBS) (42.3956° N, 85.3749° W; elevation 288 m asl) in southwestern Michigan, USA. This site is a part of the Great Lakes Bioenergy Research Center (www.glbrc.org) and is a Long-term Ecological Research site (www.lter.kbs.msu.edu). Soils are mesic Typic Hapludalfs developed on glacial outwash^54^ with high sand content (76% in the upper 150 cm) intermixed with silt-rich loess in the upper 50 cm^55^. The water table lies approximately 12–14 m below the surface. The climate is humid temperate with a mean annual air temperature of 9.1 °C and annual precipitation of 1005 mm, 511 mm of which falls between May and September (1981**–**2010)^56,57^.

The BCSE was established as a randomized complete block design in 2008 on preexisting farmland. Prior to BCSE establishment, the field was used for grain crop and alfalfa (*Medicago sativa* L.) production for several decades. Between 2003 and 2007, the field received a total of ~ 300 kg P ha^−1^ as manure, and the southern half, which contains one of four replicate plots, received an additional 206 kg P ha^−1^ as inorganic fertilizer.

The experimental design consists of five randomized blocks each containing one replicate plot (28 by 40 m) of 10 cropping systems (treatments) (Supplementary Fig. S1; also see Sanford et al.^58^). Block 5 is not included in the present study. Details on experimental design and site history are provided in Robertson and Hamilton^57^ and Gelfand et al.^59^. Leaching of P is analyzed in six of the cropping systems: (i) continuous no-till corn, (ii) switchgrass, (iii) miscanthus, (iv) a mixture of five species of native grasses, (v) a restored native prairie containing 18 plant species (Supplementary Table S1), and (vi) hybrid poplar.

### Agronomic management

Phenological cameras and field observations indicated that the perennial herbaceous crops emerged each year between mid-April and mid-May. Corn was planted each year in early May. Herbaceous crops were harvested at the end of each growing season with the timing depending on weather: between October and November for corn and between November and December for herbaceous perennial crops. Corn stover was harvested shortly after corn grain, leaving approximately 10 cm height of stubble above the ground. The poplar was harvested only once, as the culmination of a 6-year rotation, in the winter of 2013–2014. Leaf emergence and senescence based on daily phenological images indicated the beginning and end of the poplar growing season, respectively, in each year.

Application of inorganic fertilizers to the different crops followed a management approach typical for the region (Table 1). Corn was fertilized with 13 kg P ha^−1^ year^−1^ as starter fertilizer (N-P-K of 19-17-0) at the time of planting and an additional 33 kg P ha^−1^ year^−1^ was added as superphosphate in spring 2015. Corn also received N fertilizer around the time of planting and in mid-June at typical rates for the region (Table 1). No P fertilizer was applied to the perennial grassland or poplar systems (Table 1). All perennial grasses (except restored prairie) were provided 56 kg N ha^−1^ year^−1^ of N fertilizer in early summer between 2010 and 2016; an additional 77 kg N ha^−1^ was applied to miscanthus in 2009. Poplar was fertilized once with 157 kg N ha^−1^ in 2010 after the canopy had closed.

### Sampling of subsurface soil water and soil for P determination

Subsurface soil water samples were collected beneath the root zone (1.2 m depth) using samplers installed at approximately 20 cm into the unconsolidated sand of 2Bt2 and 2E/Bt horizons (soils at the site are described in Crum and Collins^54^). Soil water was collected from two kinds of samplers: Prenart samplers constructed of Teflon and silica (http://www.prenart.dk/soil-water-samplers/) in replicate blocks 1 and 2 and Eijkelkamp ceramic samplers (http://www.eijkelkamp.com) in blocks 3 and 4 (Supplementary Fig. S1). The samplers were installed in 2008 at an angle using a hydraulic corer, with the sampling tubes buried underground within the plots and the sampler located about 9 m from the plot edge. There were no consistent differences in TDP concentrations between the two sampler types. Beginning in the 2009 growing season, subsurface soil water was sampled at weekly to biweekly intervals during non-frozen periods (April–November) by applying 50 kPa of vacuum to each sampler for 24 h, during which the extracted water was collected in glass bottles. Samples were filtered using different filter types (all 0.45 µm pore size) depending on the volume of leachate collected: 33-mm dia. cellulose acetate membrane filters when volumes were less than 50 mL; and 47-mm dia. Supor 450 polyethersulfone membrane filters for larger volumes. Total dissolved phosphorus (TDP) in water samples was analyzed by persulfate digestion of filtered samples to convert all phosphorus forms to soluble reactive phosphorus, followed by colorimetric analysis by long-pathlength spectrophotometry (UV-1800 Shimadzu, Japan) using the molybdate blue method^60^, for which the method detection limit was ~ 0.005 mg P L^−1^.

Between 2009 and 2016, soil samples (0–25 cm depth) were collected each autumn from all plots for determination of soil test P (STP) by the Bray-1 method^61^, using as an extractant a dilute hydrochloric acid and ammonium fluoride solution, as is recommended for neutral to slightly acidic soils. The measured STP concentration in mg P kg^−1^ was converted to kg P ha^−1^ based on soil sampling depth and soil bulk density (mean, 1.5 g cm^−3^).

### Sampling of water samples from lakes, streams and wells for P determination

In addition to chemistry of soil and subsurface soil water in the BCSE, waters from lakes, streams, and residential water supply wells were also sampled during 2009–2016 for TDP analysis using Supor 450 membrane filters and the same analytical method as for soil water. These water bodies are within 15 km of the study site, within a landscape mosaic of row crops, grasslands, deciduous forest, and wetlands, with some residential development (Supplementary Fig. S2, Supplementary Table S2). Details of land use and cover change in the vicinity of KBS are given in Hamilton et al.^48^, and patterns in nutrient concentrations in local surface waters are further discussed in Hamilton^62^.

### Leaching estimates, modeled drainage, and data analysis

Leaching was estimated at daily time steps and summarized as total leaching on a crop-year basis, defined from the date of planting or leaf emergence in a given year to the day prior to planting or emergence in the following year. TDP concentrations (mg L^−1^) of subsurface soil water were linearly interpolated between sampling dates during non-freezing periods (April–November) and over non-sampling periods (December–March) based on the preceding November and subsequent April samples. Daily rates of TDP leaching (kg ha^−1^) were calculated by multiplying concentration (mg L^−1^) by drainage rates (m^3^ ha^−1^ day^−1^) modeled by the Systems Approach for Land Use Sustainability (SALUS) model, a crop growth model that is well calibrated for KBS soil and environmental conditions. SALUS simulates yield and environmental outcomes in response to weather, soil, management (planting dates, plant population, irrigation, N fertilizer application, and tillage), and genetics^63^. The SALUS water balance sub-model simulates surface runoff, saturated and unsaturated water flow, drainage, root water uptake, and evapotranspiration during growing and non-growing seasons^63^.

The SALUS model has been used in studies of evapotranspiration^48,51,64^ and nutrient leaching^20,65–67^ from KBS soils, and its predictions of growing-season evapotranspiration are consistent with independent measurements based on growing-season soil water drawdown^53^ and evapotranspiration measured by eddy covariance^68^. Phosphorus leaching was assumed insignificant on days when SALUS predicted no drainage. Volume-weighted mean TDP concentrations in leachate for each crop-year and for the entire 7-year study period were calculated as the total dissolved P leaching flux (kg ha^−1^) divided by the total drainage (m^3^ ha^−1^).

One-way ANOVA with time (crop-year) as the fixed factor was conducted to compare total annual drainage rates, P leaching rates, volume-weighted mean TDP concentrations, and maximum aboveground biomass among the cropping systems over all seven crop-years as well as with TDP concentrations from local lakes, streams, and groundwater wells. When a significant (α = 0.05) difference was detected among the groups, we used the Tukey honest significant difference (HSD) post-hoc test to make pairwise comparisons among the groups. In the case of maximum aboveground biomass, we used the Tukey–Kramer method to make pairwise comparisons among the groups because the absence of poplar data after the 2013 harvest resulted in unequal sample sizes. We also used the Tukey–Kramer method to compare the frequency distributions of TDP concentrations in all of the soil leachate samples with concentrations in lakes, streams, and groundwater wells, since each sample category had very different numbers of measurements.

### Compliance statement

The conduct of this research complied with the IUCN Policy Statement on Research Involving Species at Risk of Extinction and the Convention on the Trade in Endangered Species of Wild Fauna and Flora.

## Supplementary Information

Supplementary Information.

## Data Availability

The data that support the findings of this study are available from the corresponding author upon reasonable request.
