# Supplementary material for: Phosphorus availability and leaching losses in annual and perennial cropping systems in an upper US Midwest landscape
Source: Sci Rep. 2021 Oct 13;11:20367. doi: 10.1038/s41598-021-99877-7 (PMC8514564; doi:10.1038/s41598-021-99877-7)
Supplement: Supplementary file 1 — Supplementary Information. [file 41598_2021_99877_MOESM1_ESM.docx]

**Supplementary materials**

**Phosphorus availability and leaching losses in annual and perennial cropping systems in an upper US Midwest landscape**

Mir Zaman Hussain^1,2,*^, Stephen K. Hamilton^1,2,3,4^, G. Philip Robertson^1,2,5^ and Bruno Basso^1,2,6^

^1^ W.K. Kellogg Biological Station, Michigan State University, Hickory Corners, MI 49060, USA

^2^ Great Lakes Bioenergy Research Center, Michigan State University, East Lansing, MI 48824, USA

^3^ Department of Integrative Biology, Michigan State University, East Lansing, MI 48824, USA

^4^ Cary Institute of Ecosystem Studies, Millbrook, NY 12545, USA

^5^ Department of Plant, Soil, and Microbial Sciences, Michigan State University, East Lansing, MI 48824, USA

^6^ Department of Earth and Environmental Sciences, Michigan State University, East Lansing, MI 48824, USA

*Corresponding author (mirzamanhussain@gmail.com)

Table S1 Plant species composition in the restored prairie cropping system

|  | Species | Taxa |
| --- | --- | --- |
| 1 | *Elymus canadensis* | Grass |
| 2 | *Sorghastrum nutans* (L.) Nash ex Small | Grass |
| 3 | *Schizacyrium scoparium* (Michx.) Nash | Grass |
| 4 | *Andropogon gerardii* | Grass |
| 5 | *Desmodium canadense* | Forb |
| 6 | *Lespedeza capitate* | Forb |
| 7 | *Baptisia lacteal* var. lacteal | Forb |
| 8 | *Rudbeckia hirta* L. | Forb |
| 9 | *Anemone canadensis* L. | Forb |
| 10 | *Asclepias tuberosa* L. | Forb |
| 11 | *Monarda fistulosa* L. | Forb |
| 12 | *Silphium perfoliatum* L. | Forb |
| 13 | *Ratibida pinnata* (Vent.) Barnh. | Forb |
| 14 | *Solidago rigida* L. | Forb |
| 15 | *Solidago speciose* L. | Forb |
| 16 | *Aster novae-angliae* L. | Forb |
| 17 | *Koeleria cristata* | Grass |
| 18 | *Panicum virgatum* var. Southlow | Grass |

Table S2. Description of lakes, streams and wells in SW Michigan from where water was sampled for TDP concentrations. The wells, streams and some lakes were sampled on multiple dates.

| Sampling sites | Coordinates (latitude, longitude) | Township/County | |
| --- | --- | --- | --- |
| Crooked Lake | N42.491600°, W85.429100° | | Delton/Barry |
| Douglas Lake | N42.356270°, W85.367126° | | Augusta/Kalamazoo |
| Duck Lake | N42.408419°, W85.381941° | | Ross/Kalamazoo |
| Eagle Lake | N42.322815°, W85.323590° | | Charleston/Kalamazoo |
| Fair Lake | N42.489358°, W85.330371° | | Hickory Corners/Barry |
| Gilkey Lake | N42.481601°, W85.360072° | | Delton/Barry |
| Gull Lake | N42.402564°, W85.416145° | | Ross/Kalamazoo/Barry |
| Little long Lake | N42.422894°, W85.443878° | | Prairieville/Barry |
| Miller Lake | N42.403309°, W85.433563° | | Richland/Kalamazoo |
| Mud Lake | N42.481412°, W85.390568° | | Delton/Barry |
| Pleasant Lake | N42.491216°, W85.385134° | | Delton/Barry |
| Stony Lake | N42.377773°, W85.345235° | | Ross/Barry |
| Three Lakes | N42.351981°, W85.431972° | | Richland/Kalamazoo |
| Whitford Lawler Lake | N42.310440°, W85.356943° | | Charleston/Kalamazoo |
| Windmill Pond, KBS | N42.403601°, W85.400413° | | Ross/Barry |
| Wintergreen Lake | N42.397844°, W85.384502° | | Ross/Barry |
| Augusta Creek | N42.361439°, W85.354208° | | Augusta/Kalamazoo |
| Gull Creek | N42.301287°, W85.399527° | | Galesburg/Kalamazoo |
| Kalamazoo River | N42.676690°, W86.215310° | | Multiple |
| Prairieville Creek | N42.427616°, W85.430082° | | Prairieville/Barry |
| Stony Creek | N42.360425°, W85.310606° | | Ross/Kalamazoo/Barry |
| KBS wells | N42.409646°, W85.392782° | | Ross/Kalamazoo |

Figure S1. Experimental layout of GLBRC Biofuel Cropping System Experiment (BCSE) showing the distribution of treatments, and position of soil water samplers (suction lysimeters) in the plots.

**
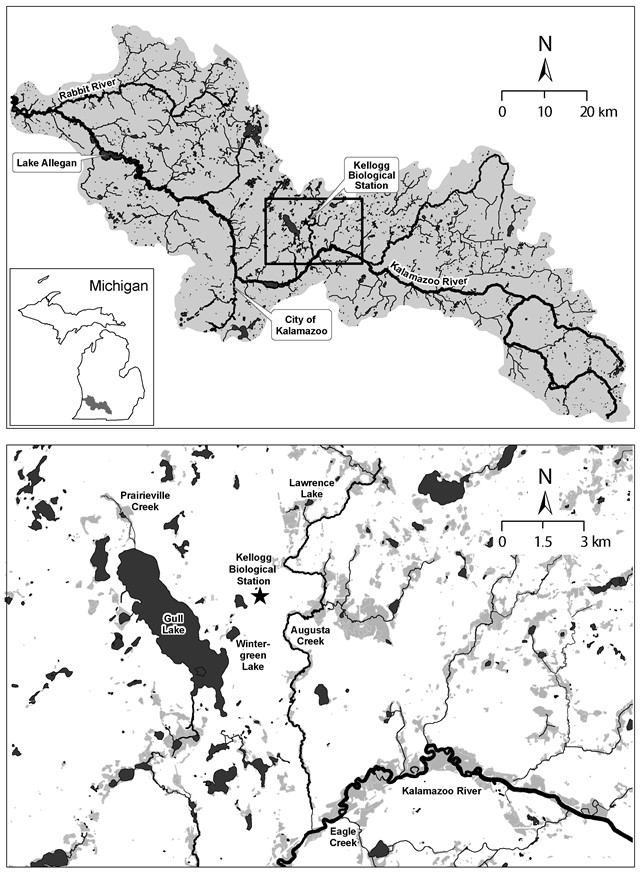
**

Figure S2. Location of the study area within the Kalamazoo River watershed (using ArcGIS software) in southwestern Michigan (Top panel); enlargement of the inset from top panel showing the area in the vicinity of the cropping systems where streams, lakes, and residential water supply wells were sampled (Bottom panel) for comparison with leachate concentrations (only selected water bodies are labeled).
